# Calm and smart? A selective review of meditation effects on decision making

**DOI:** 10.3389/fpsyg.2015.01059

**Published:** 2015-07-24

**Authors:** Sai Sun, Ziqing Yao, Jaixin Wei, Rongjun Yu

**Affiliations:** ^1^Department of Psychology and Center for Studies of Psychological Application, School of Psychology, South China Normal UniversityGuangzhou, China; ^2^School of Economics and Management and Scientific Laboratory of Economic Behaviors, South China Normal UniversityGuangzhou, China

**Keywords:** meditation, decision making, empathy, prosocial behavior, neuroimaging

## Abstract

Over the past two decades, there has been a growing interest in the use of meditation to improve cognitive performance, emotional balance, and well-being. As a consequence, research into the psychological effects and neural mechanisms of meditation has been accumulating. Whether and how meditation affects decision making is not yet clear. Here, we review evidence from behavioral and neuroimaging studies and summarize the effects of meditation on social and non-social economic decision making. Research suggests that meditation modulates brain activities associated with cognitive control, emotion regulation and empathy, and leads to improved non-social and social decision making. Accordingly, we propose an integrative model in which cognitive control, emotional regulation, and empathic concern mediate the effects of meditation on decision making. This model provides insights into the mechanisms by which meditation affects the decision making process. More evidence is needed to test our explanatory model and to explore the function of specific brain areas and their interactive effects on decision making during meditation training.

If we are to make peace in the world, we must first make peace in ourselves.

—The Dalai Lama

There is a growing body of evidence suggesting that interventions including regular physical exercise (Scully et al., 1998; Hassmén et al., 2000), cognitive behavior therapy (Beck, 1993), and ancient contemplative practices (Rice, 2001; Astin et al., 2003; Hill et al., 2006) leads to a range of positive psychological outcomes such as improved cognitive performance, enhanced emotional regulation, and even plasticity-related alterations in the brain. In particular, one type of contemplative practice, meditation, has attracted wide attention from both psychologists and neuroscientists over the past two decades due to a growing appreciation for its ability to affect cognition, emotion, and decision making.

There are various definitions of meditation depending on what main interventions are emphasized. In general, meditation is defined as a broad variety of practices designed to cultivate emotional balance and psychological well-being, including relaxation, the observation of one’s own inner or outer experiences, and the intentional self-regulation of attention (Lutz et al., 2008b; Slagter et al., 2011; Awasthi, 2012). There are many forms of meditation practice such as mindfulness meditation, concentrative meditation, transcendental meditation, Buddhist meditation, and others (Cahn and Polich, 2006; Travis and Shear, 2010). In the current review, for the most part we focus on the literature regarding mindfulness meditation, and sometimes also compassion meditation and loving-kindness meditation. Mindfulness meditation refers to a broad range of practices based on promoting a non-judgmental and non-reactive state of awareness that may improve one’s ability to modify automatic behaviors in the long run (Kabat-Zinn, 2003). Compassion meditation focuses one’s awareness mainly on alleviating the suffering of all other sentient beings, and the central point of loving-kindness meditation is a loving and kind concern for the well-being of oneself and others (Hofmann et al., 2011). Among all types of meditation, these three types are most common and most studied in research on meditation and human decision making (Chambers et al., 2008; Hofmann et al., 2011; Chiesa et al., 2013).

Evidence from behavioral studies has provided support for potential applications of meditation. In particular, a 3-month meditation retreat has been found to be associated with decreased variability in attentional processing of target tones, suggesting improved sustained attention (Lutz et al., 2009b). Also, in a 10-day program in mindfulness meditation, individuals showed decreased reaction time on an internal switching task and better performance in the Digit Span Backward subscale, suggesting a greater capacity for sustained attention, working memory, and executive function (Chambers et al., 2008). From other perspectives, researchers also found that compassion-focused meditation may increase happiness as well as decrease worry and emotional suppression (Jazaieri et al., 2014), and general meditation training may reduce emotional interference from unpleasant pictures (Ortner et al., 2007).

Notably, previous research mainly illustrates the impacts of meditation on basic emotions and cognitive functions such as attention, memory, and executive function. Beyond emotion and cognition, individuals also need to make decisions in situations involving complex social interactions (Sanfey, 2007). Decision making can be regarded as the thought processes during which a judgment or course of action is identified and selected from several alternative possibilities based on one’s values and preferences (Rilling and Sanfey, 2011). The process of decision-making is often characterized by a competition between reflection and intuition. Based on existing literature (Fehr and Camerer, 2007; Sanfey, 2007; Rilling and Sanfey, 2011), we divide decision making into non-social and social categories. Non-social decision-making research focuses on individual decisions that are made purely based on one’s own beliefs, values, and preferences, whereas research on social decision making focuses on interactive decisions that are made based on the concomitant choices and preferences of others (Fehr and Camerer, 2007; Sanfey, 2007; Rilling and Sanfey, 2011). It is not known, however, whether meditation-related experience can facilitate non-social and social decision making.

We postulate that the effects of meditation may not be limited to those aspects of cognition and emotion that are prerequisites of high-level decision making, but can also extend to decision-making processes. In particular, recent evidence has suggested that meditation may play a role in reducing economic decision biases, and enhancing the empathy, compassion, and altruism involved in social decisions (Birnie et al., 2010; Leiberg et al., 2011; Klimecki et al., 2012). Also, clinical evidence has demonstrated that meditation can be a useful tool to reduce substance abuse, alcohol addiction, and the craving to smoke (Breslin et al., 2002; Zgierska et al., 2009; Fernandez et al., 2010; Westbrook et al., 2013). These disorders are associated with impulsive behaviors (e.g., taking risks) and suboptimal decision making (Keng et al., 2011; Sedlmeier et al., 2012; Carim-Todd et al., 2013). The aforementioned behavioral findings have indicated a potential role of meditation on improving decision making in both social and non-social conditions.

As neuroimaging techniques advance, it becomes possible to study changes in the brain that occur long-term meditation. Several recent studies have provided evidence of meditation-dependent cortical plasticity, demonstrating that, compared to non-mediators, long-term meditators show long-lasting changes in the brain, such as increased cortical thickness in the prefrontal cortex and right anterior insula, greater gray matter concentration in the right insula, and increased gray matter density in the brain stem (Lazar et al., 2005; Hölzel et al., 2008; Vestergaard-Poulsen et al., 2009). Beyond brain variations, researchers also have observed increased neural activity during meditation in the dorsolateral prefrontal cortex (DLPFC), parietal cortex, hippocampus and para-hippocampus, temporal lobe, striatum, and anterior cingulate cortex (ACC) during meditation, suggesting a crucial role of meditation in cognitive control, memory processing, conflict monitoring, and reward processing (Lazar et al., 2000). Taken together, these findings have provided further neural evidence for meditation which may influence decision making via changes in the brain regions involved in reward processing, cognitive control, and emotion management.

Both behavioral and neuroimaging studies have provided new insights into the psychological function of meditation on decision making. However, no systematic review has yet integrated the evidence of these psychological effects and underlying mechanisms of meditation on decision making. In business, decision making is one of the central activities of management and is critically important for the implementation of ideas (Simon, 1987). For individuals, families and organizations making good decisions can lead to happiness (Hsee et al., 2008) and greater achievement (Shen et al., 2015). Ineffective decisions may lead to regret (Coricelli et al., 2007; Van Dijk and Zeelenberg, 2007), pain (Frantsve and Kerns, 2007) and even mental disorders (Goudriaan et al., 2005). Thus, it is of great significance to systematically review the potential effects of meditation on decision making and the neural mechanisms of these effects.

Here, we limit our review to published, peer-reviewed and empirical studies that assessed psychological outcomes of meditation on decision making. In particular, we focus on mindfulness, loving-kindness, and compassion meditation techniques, and review their influences on non-social economic and social decision making. The literature search was performed using the main keywords “mindfulness meditation,” “loving-kindness meditation,” “compassion meditation” from the electronic databases Google scholar, PubMed, Springer, ProQuest, PsycINFO, and Elsevier. We chose these databases because they include almost the whole literature related to mental health, medicine, psychology, and neuroscience. Next, we further restricted our keywords to “decision making,” “decisions,” or specific topics such as “decision bias,” “gambling,” “prosocial,” or “altruism.” Beyond articles from the main database, we also carefully identified citations from the chosen articles. Our review was restricted to English-language journal articles over the past two decades (1995–2015). There were 55 studies that met our criteria when searching with these keywords. Of these, we included only original research with a control group and a specific technique of meditation. Case studies, correlation studies, original research without control group, and any reviews or abstracts were excluded. In total, 13 studies were included in this review. We aim to (1) summarize the psychological effects of meditation on social and non-social decision making based on selected literature, (2) discuss the psychological and neural mechanisms of meditation with regard to how they impact the decision process, and (3) address major challenges encountered and directions for future studies. We hope that our review will provide some novel ideas for future research on the application of meditation to improve personal judgments, decision making, organizational behavior, and management.

## The Effects of Meditation on Non-Social Economic Decision Making

In the domain of non-social decision making, most researchers have utilized paradigms developed in game theory and behavioral economics to investigate economic preferences and decision biases in both personal and interactive situations (e.g., reward anticipation, risk taking, compulsive gambling, decision biases; Lakey et al., 2007a; Kiken and Shook, 2011; Kirk et al., 2011; Leiberg et al., 2011; Hafenbrack et al., 2014). One prominent dual-process theory has been proposed by Kahneman and Frederick (2002) to explain personal judgments and decision bias. They argued that mental processes are divided into two distinct categories based on whether they operate automatically or in a controlled, intentional fashion. Generally, decision biases are induced by instantiating controlling difficulties or emotional interference. Here, we review studies investigating the influence of mindfulness meditation on non-social decision-making processes including risk taking, impulsive gambling, negativity bias, and sunk cost bias (see **Table 1**).

**Table 1:** Summary of studies of meditation on decision making.

Psychological effects	Reference	Interventions	Samples	Psychological tasks and main scales	Design	Main findings (compared to matched controls) and effect sizes
**Studies of meditation on non-social economic decision making**			
Risk taking decisions	Lakey et al. (2007a)	Meditation & control (study 2): distinguished by dispositional state test (MAAS); without specific meditation manipulations	Mindfulness & Control (study2): n = 309 (age: 19.23 ± 1.31 years)	(1) Georgia gambling task; (2) Iowa gambling task; (3) DIGS, DSM-IV, MAAS,SCS	CT	(1) Reduced severity of gambling problems; (2) Increased adaptability of decision making
Impulsive gambling	Alfonso et al. (2011)	Mindfulness: 7-week mindfulness training (14 sessions, 60 min long, twice weekly, on two different days); Control: standard community treatment	Mindfulness: *n* = 18 Control: *n* = 16	Iowa gambling task	Pre-post design; CT	(1) Improved performance on decision-making; (2) Reduced decision-making deficits in polysubstance abusers
Negativity bias	Kiken and Shook (2011)	Mindfulness: 15-min instructional mindful breathing; Control: instructional unfocused attention or mind wandering	Mindfulness and Control: *n* = 175 (age: 19.6 ± 2.4 years)	(1) Bean Fest paradigm; (2) PANAS, FES, MAAS	RCT	(1) Reduced negativity bias; (2) Increased positive affect Effect size: $\eta_{p}^{2}$ = 0.023
Sunk-cost bias	Hafenbrack et al. (2014)	Mindfulness (study 2a): 15-min focused-breathing meditation exercise; Control: mind-wandering induction to think of whatever came to mind	Mindfulness and Control (study2a ): *n* = 57 (age: 19.40 ± 1.10 years)	(1) Sunk-cost decision task; (2) PANAS, MAAS, DMCI, SES	RCT	(1) Decreased negative affect; (2) Decreased sunk-cost bias Effect size: φ = 0.35
Negativity bias	Kiken and Shook (2014)	All participants listened to a standardized 10-min audio recording (study 2). Mindfulness: received instruction in a mindful breathing meditation; Control: received instruction to let their minds wander freely	Mindfulness and Control (study 2): *n* = 102 (age: 21.00 ± 3.73 years)	(1) Thought valence: a common thought listing procedure; (2) MAAS	RCT	(1) Mindfulness is associated with less negatively weighted thoughts, but is not directly related to positively weighted thoughts. (2) Attenuate thoughts that emphasize negativity but not those that emphasize positivity. Effect size: $\eta_{p}^{2}$ = 0.86
**Studies of meditation on social decision making**			
Fairness	Kirk et al. (2011)	Meditators: meditation experience (9.5 ± 7.8 years); Control: non-meditators	Meditators: *n* = 26 (age: 40.4 ± 10.4 years ) Control: *n* = 40 (age: 36.8 ± 10.1 years)	(1) Ultimate game (2) MAAS, KIMS	CT fMRI scan	(1) Meditators accept more unfair offers than controls; (2) Different network of brain when assessing unfairness in anterior/posterior insula, DLPFC, ACC, and thalamus
Fairness	McCall et al. (2014)	Meditation: full-time meditation retreat for at least 3 years; Control: no relative practice	Meditation: *n* = 18 (age: 54.3 ± 5.8 years) Control: *n* = 15 (age: 54.3 ± 5.8 years)	(1) A dictator game with second party punishment (2PP), third-party punishment (3PP), and third party punishment and recompense (3PR). (2) Emotional questionnaire (3) Fairness questionnaire	CT	(1) Less anger and punishment in response to unfairness; (2) More compensation of victims in response to fairness violations
Altruism	Weng et al. (2013)	Compassion: 30 min compassion training per day for 2 weeks; Control: matched reappraisal training	Compassion: *n* = 20 (age: 21.9 years) Control: *n* = 21 (age: 22.5 years)	Redistribution game	CT fMRI Behavior	(1) Increased altruistic redistribution of funds to a victim; Effect size: *d* = 0.65 (2) Altered activation in brain regions including the IPC and DLPFC
Prosocial behavior	Reb et al. (2010)	Loving-kindness: review a loving-kindness meditation audio clip lasts 8 min; Control: review a neutral audio clip lasts 8 min	Loving-kindness and Control: *n* = 49	(1) Dictator game; (2) Positive feelings on a 5-point likert scale	RCT Behavior	(1) More distribution of money to the counterpart; Effect size: $\eta_{p}^{2}$ = 0.08 (2) Positive feelings toward the counterpart Effect size: $\eta_{p}^{2}$ = 0.17
Prosocial behavior	Leiberg et al. (2011)	Compassion: a 1-day training lasts 6-h; Memory control: a 1-day training last 1-hour	Compassion: *n* = 27 (age: 24.74 ± 4.22 years) Control: *n* = 32 (age: 22.66 ± 3.8 years)	(1) Zurich prosocial game; (2) Sociodemographic questions online; (3) TAS,BDI, CLS	RCT	(1) Enhanced prosocial behavior; Effect size: $\eta_{p}^{2}$ = 0.21 (2) Increased positive mood and compassionate feelings and decreased negative mood Effect size: $\eta_{p}^{2}$ = 0.30
Prosocial behavior	Condon et al. (2013)	Meditation: 8-week study on meditation; Control: no intervention	Meditation and control: *n* = 39 (age: 25.23 ± 4.66 years)	Cognitive ability test on suffering	RCT	Increased altruistic behavior Effect size: φ = 0.36
Intergroup bias	Kang et al. (2014)	Loving-kindness: an hour practice per week for 6 weeks; 40 min discussion per week for 6 weeks; Waitlist control: have no any contact with the instructor or course materials until the posttest	Lovingkindness and control: *n* = 101 (age: 25.20 ± 5.20 years)	(1) Implicit association test (IAT) (2) MSTI, PSS	RCT	Decreased implicit bias toward blacks and homeless people with loving-kindness practice
Intergroup bias	Lueke and Gibson (2014)	Mindfulness: a 10-min mindfulness recording; Control: a control recording	Mindfulness and control: *n* = 72 (age:18∼23 years)	(1) IAT; (2) MRWP, MAAS	RCT	(1) Less implicit racial bias; Effect size: $\eta_{p}^{2}$ = 0.06 (2) Less implicit age bias; Effect size: $\eta_{p}^{2}$ = 0.06

*ACC, Anterior Cingulate Cortex; BDI, Beck Depression Inventory; CLS, Compassionate Love Scale; CT, Controlled trial; DLPFC: Dorsolateral Prefrontal Cortex; DMCI: Decision-Making Competence Inventory; DIGS: Diagnostic Interview for Gambling Severity; DSM-IV: Diagnostic and Statistical Manual of Mental Disorders, Fourth Edition; FES: Future Events Scale; IPC: Inferior Parietal Cortex; KIMS: Kentucky Inventory of Mindfulness Skills; MAAS: Mindful Attention Awareness Scale; MRWP: Motivation to Respond without Prejudice Scale; MSTI: multi-source interference task; PANAS: Positive Affect and Negative Affect Scales; PSS: perceived stress scale; RCT: Randomized controlled trial; SCS: Self Control Scale; SES: Self-esteem Scale; TAS: Toronto Alexithymia Scale.*

In general, risk-taking refers to a tendency to engage in behaviors that can be harmful or dangerous, but which meanwhile create an opportunity for positive outcomes. In particular of the economic domain, risk-taking is defined as a disposition to gamble after loss, increased preoccupation with gambling, enhanced necessity to take risks, and more restlessness when losing money (Winters et al., 2002). Such decision-making deficits are generally reflected in gambling tasks like the Georgia Gambling Task (GGT), which measures overconfidence and willingness to take risks (Goodie, 2003), and the Iowa Gambling Task (IGT) which assesses risk preference in relation to uncertainty, reward, and penalties (Bechara et al., 1994; Lakey et al., 2007b). Using both the GGT and the IGT with a large sample of college students (*N* = 309), Lakey et al. (2007a) explored the influence of trait mindfulness on risk-taking behavior. They found that increased dispositional mindfulness predicted a reduced severity of gambling outcomes and increased adaptability of decision making. Alfonso et al. (2011) first investigated the effects of meditation on risk taking among 18 abstinent polysubstance abusers (who were considered to have clinically significant deficit in executive function and decision making). These authors also found a significant beneficial effect of mindfulness meditation on response inhibition and risky decision making, suggesting a potential role of meditation for improving impulsive gambling inhibition, decision-making dysfunction, and addiction treatment.

People are highly susceptible to judgment and decision biases (Weng et al., 2013). Negativity bias is the tendency to weigh negative information, events, or emotions more heavily than the positive (Rozin and Royzman, 2001). This bias may be related to threating signals or habitual responses (Rozin and Royzman, 2001; Kiken and Shook, 2011). Using a 15-min instructional mindfulness breathing exercise, one study among 175 college students demonstrated that meditation can decrease negativity bias ($\eta_{p}^{2}$ = 0.023) and increase positive judgments in an attitude formation task (Kiken and Shook, 2011). Another study of 102 undergraduate students revealed that a standardized 10-min instruction in a mindful breathing meditation can weaken thoughts that emphasize negativity ($\eta_{p}^{2}$ = 0.86) (Kiken and Shook, 2014). The findings suggest that meditation interventions can significantly reduce negativity by precluding habitual reactions toward negative ratings or stimuli.

Sunk cost bias, also known as the sunk cost fallacy, is a tendency to continue to pursue a failing endeavor once an investment in money, effort, or time has been made (Maréchal, 2010). People often report falling victim to the sunk cost bias, even though they know that continuing is not the best choice. This bias may be related to the escalation of commitment, entrapment, anticipated regret, and loss aversion (Brockner et al., 1986; Brockner, 1992; Tversky and Kahneman, 1992; Wong and Kwong, 2007). Recently, Hafenbrack et al. (2014) investigated the short-term effects of mindfulness meditation on sunk cost biases and found that mindfulness meditation can modulate one’s temporal focus away from the future and past, and reduce negative affect, thereby decreasing the strength of the sunk cost bias (φ = 0.35).

From the above findings, we conclude that meditation-related experience can reduce impulsivity, pathological gambling, and decision biases in non-social decision making. These effects indicate a modulating role of meditation during decision making by controlling risky responses, precluding habitual actions, regulating temporal focus, and reducing negative emotions.

## The Effects of Meditation on Social Decision Making

The behavioral studies mentioned above mainly addressed irrational decisions or decision biases in non-social situations. Whether or not meditation can influence social decision making remains unclear. Here, we describe several of the most relevant studies on this topic, in which mindfulness meditation, loving-kindness meditation, and compassion meditation have been examined in terms of their effects on social decisions, such as assessments of fairness, altruism, prosocial responses, and prosocial behavior.

Assessing the fairness of a social interaction is an important aspect of prosocial behavior. Sensitivity to fairness is generally studied using the ultimatum game. In this game, two people, a proposer and a responder, are involved. The responder decides whether or not to accept or reject offers from the proposer to split a pot of money (either evenly or unevenly). If the responder accepts, both players gain accordingly. If the responder rejects the offer, neither person is paid (Crockett et al., 2010). Using an ultimatum game, it has been found that individuals who meditate are more willing to accept unfair offers compared to non-meditators. At the neural level, control participants exhibit greater activation in the anterior insula during unfair offers. Meditators display attenuated activity of the anterior insula for high-level emotional representations and increased activity of the posterior insula for low-level internal representations. This suggests that a different network of brain regions is involved among meditators to untangle negative emotional reactions (Kirk et al., 2011). Researchers also found that loving kindness meditation practitioners show less anger, less punishment, and more compensation of victims in response to fairness violations compared to controls, and this may result from the enhanced kindness to victims and cultivation of altruism with compassion meditation (McCall et al., 2014). Based on these studies, we suggest that meditation experience can help to regulate negative emotions or cultivate compassion during social decision making, leading to the acceptance of more unfair offers.

Altruism represents a motivational state to benefit others (Schwartz, 1977). Using 8-min loving-kindness meditation training, researchers have explored the effect of meditation on altruistic behavior in a dictator game. In these games, one person (the “dictator”) can unilaterally allocate any part of a given resource to others without worrying about reprisal. In one study, participants typically show empathic concern and prosocial orientation toward their counterparts ($\eta_{p}^{2}$ = 0.08), and these feelings were fully mediated by positive feelings toward others ($\eta_{p}^{2}$ = 0.17; Reb et al., 2010). Meditation experience was shown to promote more altruistic behavior (giving more of the resource to the counterpart), which is mainly modulated by the positive emotions generated during the training.

Using a redistribution task combined with neuroimaging techniques, Weng et al. (2013) investigated the neural mechanisms underlying the effects of short-term compassion meditation on altruistic behavior. During this task, participants observed a virtual circumstance in which a victim received unfair treatment. Participants could then choose to spend any amount of their own money to redistribute funds to the victim. Compared to the control group, compassion meditators were found to give more of their funds to victims ($\eta_{p}^{2}$ = 0.65), and this behavior was associated with altered activation in brain regions associated with social cognition and emotion regulation, including the inferior parietal cortex, DLPFC, and its connectivity with the nucleus accumbens. Such studies suggest that greater altruistic behavior may be elicited by increasing engagement of the neural systems associated with understanding the suffering of others, executive control, and reward processing.

In line with this, recent research has also investigated the effects of meditation on more general prosocial behavior, which covers a wide range of actions that benefit others, such as cooperation, helping, and sharing (Batson and Powell, 2003). In one study, Leiberg et al. (2011) instructed participants to navigate a virtual character through a maze to reach a treasure in a limited amount of time. This task limited the influence of reciprocity, cost, and distress, but allowed for the repeated assessment of prosocial behavior. Results demonstrated that subjects with compassion meditation training compared to those who received memory skills training showed more prosocial behavior ($\eta_{p}^{2}$ = 0.21). Additionally, the effectiveness of compassion training was further promoted by increasing positive mood and compassionate feelings and by decreasing negative mood ($\eta_{p}^{2}$ = 0.30). From such studies, we can conclude that even short-term compassion training can have a positive impact on prosocial behavior toward strangers, which relies on emotion regulation. These findings suggest one pathway by which meditation may promote prosocial behavior.

Beyond these laboratory studies, a recent study used more ecologically valid methods to investigate the effect of meditation on empathy with real-time interpersonal interactions (Condon et al., 2013). Prosocial responses were measured by whether a participant offered his or her seat to an individual with a physical disability. Results revealed that participants who had taken an 8-week course on meditation were more likely to offer up their seats than those on a waiting-list control group (*φ* = 0.36), indicating increased altruistic behavior in a real-life situation following a meditation intervention.

Furthermore, researchers have also explored the impact of mediation on implicit and explicit biases. Using an Implicit Association Test (IAT), Kang et al. (2014) found a significant decrease of implicit biases toward Blacks and homeless people with a 6-week loving-kindness practice. They suggested that loving-kindness meditation can automatically activate implicit attitudes toward different stigmatized social groups via the increase of cognitive control and decrease of psychological stress. Another study demonstrated a significant decrease of age and racial biases (both effect size: $\eta_{p}^{2}$ = 0.06) among participants who listened to a 10-min mindfulness recording relative to those who listened to a natural history. They suggested that the significant reduction of implicit biases was induced by automatic associations between mindfulness and biases (Lueke and Gibson, 2014).

In summary, the studies presented above suggest a consistent positive effect of both short- and long-term meditation on altruism, prosocial behavior, moral decision making, and intergroup bias. Meditation may facilitate such decision making by modulating executive control, reward processing, emotion regulation, and/or empathic concern involved in the decision process.

## The Mechanisms of Meditation and Their Effects on Decision Making

**FIGURE 1:**
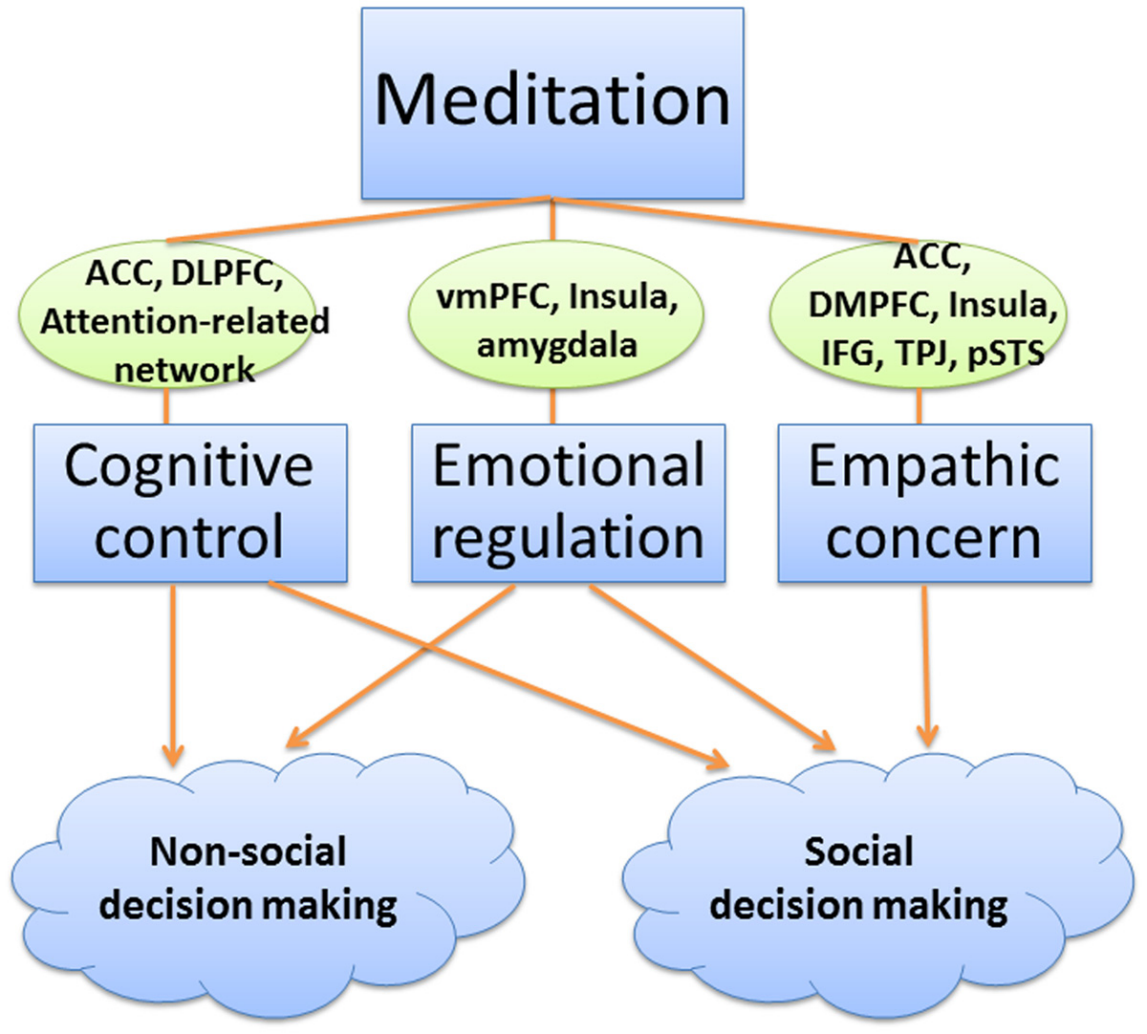
**A model for the effects of meditation on decision making.** Meditation modulates brain activities associated with cognitive control, emotion regulation and empathy, and lead to improved non-social and social decision making. Our proposed model is a combination of existed behavioral and neuroimaging findings, theoretical guidance of dual-process theory, as well as proper speculations on the underlying mechanisms. Both the specific neural findings and the modulation effects of cognitive control, emotional regulation, and empathic concern between meditation and decision making are supported by extant research literature. However, more evidence is needed to explore the function of specific brain areas and their interaction effects on decision making during meditation training. ACC: anterior cingulate cortex; DLPFC: dorsolateral prefrontal cortex; dmPFC: dorsomedial prefrontal cortex; IFG: inferior frontal gyrus; pSTS: posterior superior temporal sulcus; TPJ: temporo-parietal junction; vmPFC: ventromedial prefrontal cortex.

Overall, the research presented above suggests that meditation interventions can promote good decision making, reduce decision bias, and improve altruistic and prosocial behaviors. Next, we introduce one unifying theoretical framework for the effects of meditation on decision making (see **Figure 1**). Our proposed model is based on dual-process theory, in which automatic (or intuitive) and deliberate (or reflective) processes are considered to be two separate components of decision making. This theory is widely accepted by researchers to explain decision-making processes and the relationship between cognition, emotion, and decision making. Here, we extend the dual-process model to explain the effects of meditation on non-social economic and social behaviors and the mechanisms of those effects. We additionally consider the mediating role of empathy in social decision making based on the two studies discussed above (Leiberg et al., 2011; Weng et al., 2013).

### Cognitive Control Promotes Reflective Judgments

Conceptually, meditation places an emphasis on observing particular aspects of inner or outer experience, intentional self-regulation of attention, and the promotion of non-judgmental and non-reactive awareness. This collection of processes is similar to cognitive control, which has been defined as the selection of goal-relevant information, performance monitoring, and the storage and manipulation of information in working memory, from which individuals can flexibly adapt their behavior to pursue an internal goal (Slagter et al., 2011). Behavioral evidence suggests that meditation-related interventions can increase sensitivity to sensations, thoughts, and feelings, and lead to more sustained attention (MacLean et al., 2010), cognitive flexibility (Moore and Malinowski, 2009), and working memory (van Vugt and Jha, 2011). Meditation can also decrease rumination (Ramel et al., 2004), negative automatic thinking (Frewen et al., 2008), and habitual responding (Wenk-Sormaz, 2005). Thus, with short- or long-term meditation interventions, individuals can improve their cognitive control capabilities (Chambers et al., 2008). Thus, we propose that meditation can help decision-makers to reach conclusions with a more reflective consideration of their values and objectives, allowing them to better differentiate between relevant and irrelevant information, maintain goal awareness, and reduce irrational behaviors. In addition, with enhanced cognitive control and reflective thinking, people who meditate may be able to reduce some habitual tendencies such as engaging in risky decisions, obsessing about past or future considerations, and reacting automatically in a negative or undue manner. Thus, we propose that meditation can improve decision-making abilities with enhanced self-monitoring and cognitive control.

Recent neuroimaging studies have provided new insights into the potential neural mechanisms by which meditation affects decision making. Specifically, these studies have suggested positive effects of meditation on attention, memory, response inhibition, self-regulation, and reward processing. In particular, Brefczynski-Lewis et al. (2007) found that the association between meditation and activation in the neural networks involved in sustained attention could be represented by an inverted U-shaped curve. Similarly, other researchers have demonstrated that expert meditators exhibit reduced brain activation in regions related to discursive thoughts and emotions (mainly in ventral attention network regions), and greater activation in regions related to response inhibition and attention (mainly prefrontal regions, basal ganglia, and sub-thalamic nuclei), suggesting that meditation practice can modify and enhance the mechanisms underlying cognitive control over automatic behaviors (also known as top–down neural activity) (Corbetta and Shulman, 2002; Ochsner et al., 2002; Aron and Poldrack, 2006; Carim-Todd et al., 2013). Moreover, Lazar et al. (2000) identified several functional brain regions that are active during meditation, such as the DLPFC, parietal cortex, temporal lobe, hippocampus and parahippocampus, striatum, and pregenual ACC. These areas are related to attention, memory, reward processing, and arousal/autonomic control. Xue et al. (2011) have also shown that even short-term meditation interventions can increase the network efficiency of the ACC, which is crucial for conflict monitoring and performance adjustment. Overall, these findings on the effects of meditation on cognitive processing on the neural level support the notion that meditation may improve several aspects of decision making. In summary, both behavioral and neural evidence of cognitive processing provide evidence that meditation affects decision making. We propose that meditation can enhance reflective decision making by improving cognitive control over habitual reactions and intuitive processing.

### Emotion Regulation Reduces Intuitive Decisions

In addition to cognitive control, meditation can also affect emotion regulation, which can play a crucial role in decision making, especially in the social domain. Emotion regulation refers to a variety of strategies applied at different points during the generation of emotional responses that influence what, when, and how emotions arise, persist, and are experienced and expressed (Gross and Thompson, 2007). Notably, during meditation there is a particular awareness and non-judgmental acceptance of the present, which may enhance one’s sensitivity to affective cues and lead to more timely emotion regulation, reactions, and hyper-vigilance (Block-Lerner et al., 2007). In such a way, meditation may modify decision making by promoting proper emotion regulation. In other words, we propose that meditation interventions may lead to better decisions by promoting better emotion regulation.

Evidence from behavioral and neuroimaging studies provide some support for the effects of meditation on decision making via emotion regulation. At the behavioral level, well-established research by Kiken and Shook (2011) has indicated that even short-term meditation interventions can reduce negativity bias and increase positive judgments. These effects, however, are mainly modulated by attention reallocation, the suppression of intuition, and executive control (Slagter et al., 2007; Kozasa et al., 2012). Research on structural brain changes associated with mindfulness have demonstrated a positive association between trait mindfulness and gray matter volume in the right anterior insula and the right amygdala, regions related to emotional/bodily states and intuitive responses. Taken together, these studies suggest that meditation may enhance decision making through the regulation of negative/positive emotions, thereby improving cognitive control over intuitive decisions.

### Empathic Concern Facilitates Social Decisions

Empathy has been associated with increased helping and social support (Coke et al., 1978). In addition to the modulating effects of cognitive control and emotion regulation on decision making, we also found a crucial role of empathy in enhancing prosocial behavior during meditation training. Conceptually, meditation interventions, and compassion meditation and loving-kindness meditation in particular, involve training in understanding the feelings of others and a focus on alleviating their suffering. Empathy also elicits other-oriented emotions depending on the perceived well-being of others (Batson et al., 2011). Behaviorally, a number of social and developmental studies have demonstrated that short-term inductions of empathic concern can motivate prosocial behavior (Batson et al., 2007). Thus, it is possible that compassion or loving-kindness meditation can improve social decisions by promoting empathy and a better understanding of others.

Evidence from neuroimaging studies provides support for the effect of meditation on pro-social decisions through increased empathy. Mascaro et al. (2013) demonstrated that an 8-week compassion intervention improved empathic accuracy which is positively correlated with neural activity in the inferior frontal gyrus and dorsomedial prefrontal cortex. Structural neuroimaging studies have demonstrated that loving-kindness and compassion meditation altered the activation of circuits previously linked to empathy (insula and ACC) and theory of mind in response to emotional stimuli (amygdala, right temporo-parietal junction, and right posterior superior temporal sulcus) (Lutz et al., 2008a). These studies provided indirect evidence of the mediating role of empathy. Using a redistribution task combined with functional magnetic resonance imaging (fMRI) techniques, Weng et al. (2013) found that empathy-related brain networks are involved in the effects of meditation on prosocial decisions, suggesting a facilitating role of empathy on social decision making after meditation interventions. The psychological effects of meditation may depend not only basic cognitive processing and emotion regulation, but also on more advanced social capabilities, such as empathy.

### Conclusions Regarding the Mechanisms of Meditation

Based on these previous studies, we propose one explanatory model for the effects of meditation on decision making that includes aspects of cognitive control, emotion regulation, and empathy. We have explored some of the neural mechanisms potentially underlying this model. We posit that the beneficial effects of meditation on decision making may be modulated by cognitive control, emotion regulation, and empathic concern, which are three important contributors to more rational decisions and prosocial behaviors. It should be noted that, in the current review, our model is mainly based on the extant relevant empirical studies. It is possible that additional mechanisms may be involved but have yet to be identified.

## Limitations and Future Directions

Several important limitations in our review are worth mentioning. First, the current review focuses on three most studied types of meditation and their influence on decision making, but there many other forms of meditation, including concentrative meditation, transcendental meditation, Buddhist meditation and others. It remains unknown whether the different types of meditation result in similar effects and changes to decision making. Future studies may compare different forms of meditation in terms of their impact. Second, although we describe three distinct processes that may potentially underlie the effects of meditation, little direct evidence for the causal role of cognitive control, emotion regulation, and/or empathy has been provided. Using fMRI, future studies can further examine how meditation modulates activity in brain regions associated with cognitive control and emotion regulation in decision-making tasks. Additionally, in future research, other techniques like transcranial direct-current stimulation and transcranial magnetic stimulation may help to explore the function of specific brain areas on decision making during meditation training. Recent studies have shown that meditation experience modulates resting-state brain activity or functional connectivity in the default mode network, ACC, insula, and attention-related networks (Brefczynski-Lewis et al., 2007; Lutz et al., 2009a; Brewer et al., 2011; Xue et al., 2011). Research also shows that stimulating DLPFC can induce similar changes (Newberg et al., 2001). Third, we only briefly mentioned the separate influence of cognitive control, emotion regulation, and empathy on decision making following meditation interventions; however, some interaction effects may also exist. Thus, we suggest our model should be interpreted with caution and used as a guide for further studies to investigate such interactions. Finally, it is worth mentioning that there are only a small number of studies (*n* = 13) on the effects of meditation on decision making. More research is needed to better our understanding of how meditation shapes social decision making.

## Conclusion

In this review, we have integrated findings on the effects of meditation on decision making, empathy, and prosocial behavior. This line of research has produced to promising data suggesting that meditation interventions may be effective in promoting good decision making and increasing prosocial behavior. However, an equally important direction for future research is to investigate the neural mechanisms underlying meditation interventions. In the present paper, we propose one explanatory model that accounts for the effects of meditation on decision making by way of changes to cognitive control, emotion regulation, and empathetic concern. This model has important implications for additional research and continues to shed light on the potential mechanisms underlying the effect of meditation on decision-making processes. More evidence is needed to test our explanatory model and explore the function of specific brain areas on decision making during meditation training. Finally, we address some limitations of the current review and indicate several future directions. This review provides a useful conceptual model of the significance of meditation for decision making in both social and non-social domains.

## Author Contributions

SS, ZY, and JW wrote the first draft of the paper. SS, ZY, and RY edited drafts and contributed intellectually to the paper. All authors read and approved the final manuscript.

## Conflict of Interest Statement

The authors declare that the research was conducted in the absence of any commercial or financial relationships that could be construed as a potential conflict of interest.
